# Links between self-regulation patterns and prosocial behavior trajectories from middle childhood to early adolescence: a longitudinal study

**DOI:** 10.3389/fpsyg.2024.1480046

**Published:** 2024-12-16

**Authors:** Carolin Ritgens, Rebecca Bondü, Petra Warschburger

**Affiliations:** ^1^Department of Developmental Psychology, Psychologische Hochschule Berlin, Berlin, Germany; ^2^Department of Psychology, University of Potsdam, Potsdam, Germany

**Keywords:** self-regulation, prosocial behavior, childhood, adolescence, trajectories

## Abstract

Prosocial behavior that conforms to social norms and serves the good of others requires particularly high self-regulatory competences, because it is often in contrast with one’s own interests. It is unknown which self-regulatory competences are particularly important for prosocial-behavior development and whether they may distinguish between children on different prosocial-behavior trajectories. This longitudinal study examined differences in self-regulatory competences, including inhibition, emotional reactivity, planning behavior, emotion regulation, working-memory updating, affective decision making, flexibility, and delay of gratification, between trajectories of prosocial behavior in 1,657 German 6- to 13-year-olds (52% female). LCGA suggested four trajectories of stable high, stable low, increasing, and decreasing prosocial behavior. MANOVAs showed differences between trajectories in inhibition and emotional reactivity at all three measurement points, as well as planning behavior at the second measurement point. Early patterns of these self-regulatory skills may help identifying children at risk for impaired long-term prosocial-behavior development and should primarily be addressed by prevention and intervention measures.

## Introduction

Prosocial behavior (PB) that conforms to social norms and serves the good of others has numerous advantages also for oneself in the long run but requires effective self-regulation (SR; Davidov et al., 2016), because it often contrasts one’s own short-term interests. However, PB is associated with long-term benefits, including a lower risk for internalizing and externalizing problems, social exclusion, as well as impaired health and well-being (Flynn et al., 2015; Tintori et al., 2021). Thus, it is of great interest to maximize PB over longer periods of time and to identify children that need help early. Different SR competences have been reliably positively related to PB. SR is a complex, multi-layered construct (Bailey and Jones, 2019) that refers to a set of interrelated competences enabling the control of one’s own behavior, cognitions, and emotions, allow individuals to effectively respond to internal and external demands, and to obtain future benefits (Bailey and Jones, 2019; Nigg, 2017; Robson et al., 2020; Vohs and Baumeister, 2004). It is, therefore, crucial to understand how different SR competences may benefit PB and which of these competences are the most relevant. It may be particularly helpful to identify differential associations between these competences and PB trajectories to understand potential causes for differences in the long-term development of PB better. Using a person-centered, longitudinal approach to carve out different trajectories and potential differences in their SR competences throughout middle childhood into early adolescence is well suited to reach these aims. This approach is valuable because it allows for identifying developmental paths that are relevant for larger groups of individuals, recognizing that there are common patterns within these groups. Integrating multiple SR measures potentially allows for identifying early problematic patterns of these competences and for deriving tailored intervention measures for children on different trajectories. It is important to do so during the childhood years, when both, PB and SR further develop (Colman et al., 2006; Tintori et al., 2021) and when children have to master multiple social and academic challenges (Eisenberg et al., 1998; Raffaelli et al., 2005). Nonetheless, studies on the associations between PB and SR competences have focused on infancy and early childhood cross-sectionally, leaving gaps in knowledge on their interrelations after early childhood. The present longitudinal study, therefore, aimed to expand the current knowledge by exploring PB trajectories over a three-year period from middle childhood into early adolescence and examining their differential associations with a broad range of SR competences. Using a longitudinal design, our study contributes to existing information on the developmental trajectories of both PB, as well as the relations of the distinct SR competences and PB trajectories at three time points across middle childhood.

PB benefits others for the sake of their welfare, whereas often requiring to relinquish own benefits, thereby reflecting a moral quality (Malti et al., 2009a; Warneken and Tomasello, 2009). It is commonly considered as intervening actions that follow observations of others’ negative states (Geraci and Franchin, 2021). Thus, PB occurs if an individual, first, recognizes a negative experience of another individual, second, determines what causes this experience, third, determines an appropriate response and, fourth, acts to intervene (Dunfield, 2014; Geraci and Franchin, 2021). PB comprises helping as a response to others’ problems, sharing as a response to material desire, comforting as a response to emotional distress, and defending as a response to an aggressor’s antisocial action (Eisenberg et al., 1998; Geraci and Franchin, 2021; Warneken and Tomasello, 2009). Understanding others’ negative states and their causes requires intuitive, automatic, and emotion-based evaluations (Geraci and Franchin, 2021). Although PB may counter one’s short-term goals, it is positively related to numerous long-term goals, including social acceptance, satisfying personal relationships, and mental health (Davidov et al., 2016). However, not all humans behave equally prosocially, and research suggests differential developmental paths (Malti and Dys, 2018). Thus, it is important to identify factors that may benefit or impede PB in the long run. PB development has been associated with a wide variety of individual (e.g., self-regulation, cognitive, behavioral and social skills, pubertal maturation) and contextual (e.g., home environment, peer influences, victimization) factors (Carlo et al., 2012; Silke et al., 2018; Warneken and Tomasello, 2009). For example, SR skills have shown associations with PB (see below) and may, therefore, help to further explain individual differences in its development.

PB development, however, is not homogeneous. Whereas infants demonstrate unbiased prosociality, this innate tendency for unconditional prosociality seems to evolve somewhen between 2.5 and 5 years of age as social preferences emerge, and children become more selective of their PB (Geraci et al., 2024). Being more aware and selective of their own PB, differences in the individual levels of PB emerge and tend to remain stable during and beyond middle childhood: Studies following participants throughout middle childhood and sometimes into adolescence consistently suggested trajectories of stable high and stable low PB (Côté et al., 2002; Flynn et al., 2015; Griese et al., 2016; Malti et al., 2013) as well as additional trajectories of stable moderate (Côté et al., 2002; Flynn et al., 2015), increasing (Malti et al., 2013), and/or decreasing (Caprara et al., 2001; Griese et al., 2016; Kokko et al., 2006; Shi et al., 2021) PB. In studies that identified multiple trajectories, most participants belonged to the stable moderate (Côté et al., 2002; Flynn et al., 2015) or stable high trajectories (Griese et al., 2016; Shi et al., 2021). Thus, individual differences in PB tend to remain stable, often into adulthood (Eisenberg et al., 2002; Côté et al., 2002; Eisenberg and Mussen, 1989; Tintori et al., 2021).

Among other factors, individual PB trajectory membership has been associated with parenting, parents’ marital status, parental support, peer support, teacher-child conflict, sympathy, and gender (Flynn et al., 2015; Lee et al., 2022; Malti et al., 2009b; Padilla-Walker et al., 2015; Shi et al., 2021). For example, being female or having greater parental and peer support were associated with membership in high PB trajectories (Flynn et al., 2015; Lee et al., 2022; Malti et al., 2013). Low overall SR was associated with low PB trajectory membership (Padilla-Walker et al., 2015). Working out exactly which SR competences are especially important for a development on the high trajectory or those SR competences putting children at risk for becoming members of a low trajectory is, therefore, important as this allows for tailoring interventions training these SR competences specifically. To date, however, no study has investigated the links between different specific SR competences and PB trajectory membership.

Due to their manifold positive consequences, acquiring SR competences is a major developmental task. During the first years of life, there is substantial growth in SR from infants that heavily depend on external regulation to children who increasingly succeed in internally monitoring their behavior, directing their attention, and regulating their emotions. Some studies show further major increases in SR particularly in early and middle childhood (Raffaelli et al., 2005), others in middle childhood and early adolescence (Barkley, 1997; Demetriou, 2000; Green et al., 1994; Memmott-Elison et al., 2020). There are individual differences in the neurobiological development of basal and complex functioning (Williams and Berthelsen, 2017), which could lead to differences in the development of cognitive, emotional, and affective SR. Therefore, it is crucial to consider the distinct aspects of SR, especially when studying SR in individuals that are still undergoing neurological development. Importantly, SR competences are also open to intervention: Factors promoting positive SR development include parental supportiveness and adaptive parenting styles (Liew et al., 2018) as well as specific intervention programs (Cleary et al., 2008; Papies and Hamstra, 2010).

Inconsistencies in findings concerning SR development can partly be explained by differences in SR competences. A multitude of different constructs, terms, and definitions exist. In its complexity, SR includes both top-down and bottom-up processes that influence each other (Nigg, 2017). For both top-down and bottom-up processes to work effectively, a range of different competences involved in these processes are required. In research on SR, the processes involved are described to integrate aspects of cognition, behavior, and affect (McClelland et al., 2015). To capture SR in its full complexity the present study, therefore, considered multiple SR competences capturing cognitive, affective, and/or behavioral aspects of SR: Cognitive aspects include inhibition, which is the ability to suppress primary behavioral impulses (Miyake et al., 2000), working-memory updating (updating), which is the ability to maintain and manipulate information over short periods of time (Miyake et al., 2000), and flexibility, which enables to shift between mental sets and external demands (Miyake et al., 2000). Affective aspects include emotional reactivity, which describes the extent to which individuals experience and show strong emotions (Calkins et al., 1999), emotion regulation, including strategies which allow for monitoring, evaluating, and modifying emotional responses (Liebermann et al., 2007), as well as affective decision-making, describing the extent to which individuals make decisions on the basis of emotions and take risks (Crone and van der Molen, 2004). Lastly, the behavioral aspects of SR include planning behavior, that allows individuals to anticipate future events, set goals, and develop steps towards these goals (Haith, 1997), and delay of gratification, the ability to forgo an immediate reward in favor of a later, more valuable reward (Bailey and Jones, 2019; Poland et al., 2016). Inhibition, updating, emotion regulation, planning behavior, and delay of gratification increase during middle childhood into (early) adolescence (Carriedo et al., 2016; Raffaelli et al., 2005; Silvers et al., 2012; Yap et al., 2007). Flexibility and affective decision-making emerge in early childhood and develop and stabilize throughout childhood and adolescence (Anderson, 2002; Crone and van der Molen, 2004). There were no age differences in emotional reactivity among 10- to 23-year-olds, suggesting relative stability in level of emotional reactivity during this age range (Silvers et al., 2012). Finally, delay of gratification levels were highest in late adolescence (Achterberg et al., 2016; Burns et al., 2021). These findings suggest that the SR competences develop at differing rates throughout childhood and adolescence. Some competences can be assumed to interact (e.g., inhibition, updating, flexibility, and emotional reactivity were all shown to correlate), others correlate only slightly (Liebermann et al., 2007). Thus, differentiating between SR competences is important (Hartung et al., 2020). Nonetheless, previous research has often only considered single or specific combinations of these competences. Therefore, little is known about which may be most important for PB.

Empirical research underscores the relevance of SR for PB: For example, SR in early childhood predicted PB in early adolescence (Liew et al., 2018) as well as from early to mid-adolescence (Carlo et al., 2012; Memmott-Elison et al., 2020), but not from mid- to late adolescence (Memmott-Elison et al., 2020). These findings suggest particularly strong effects throughout late childhood and early adolescence, when SR competences still mature and expectations to behave prosocially increase (Padilla-Walker et al., 2015; Steinberg, 2010). Regarding single SR competences, previous research showed consistent positive correlations between PB and inhibition (Moriguchi et al., 2020; O’Toole et al., 2017; Paulus et al., 2015; Yavuz et al., 2022), updating (Moriguchi et al., 2020; Traverso et al., 2020), emotion regulation (Benita et al., 2017; Moriguchi et al., 2020; Schütz and Koglin, 2022; Silke et al., 2018), planning behavior (Baumsteiger, 2017; Steinbeis and Over, 2017), as well as delay of gratification (Moriguchi et al., 2020; Paulus et al., 2015), and negative correlations with emotional reactivity (Carlo et al., 2012). Previous results were contradictory regarding flexibility (Moriguchi et al., 2020; Wilson, 2003). No correlations were found with affective decision-making (O’Toole et al., 2017).

Theoretically, process models of PB offer valuable insight into the relation between SR and PB: Among other factors, cognitive and affective regulatory processes are argued to promote PB by enabling individuals to understand the needs and feelings of others and to effectively manage their own emotional reactions, thereby preventing personal distress and allowing them to act on empathic feelings in a constructive way (Eisenberg et al., 2005; Eisenberg and Fabes, 1998). More specifically, cognitive aspects, such as updating, could enable individuals to understand and respond to others’ perspectives and needs in a prosocial manner; affective aspects, such as emotional reactivity and emotion regulation, may foster calm and thoughtful decisions, thereby reducing impulsive, self-centered reactions, while heightening one’s experience of sympathy, thereby increasing prosociality (Eisenberg et al., 2015). Likewise, Warneken and Tomasello (2009) highlight the role of children’s socio-cognitive understanding for enabling higher quality and greater quantities of PB. Further research expands the understanding of affective processes by integrating the role of emotional responses and social norms in guiding PB. Social Norms thereby act as a framework within which affective processes are integrated and acted upon, guiding individuals towards societal expectations of prosociality (Vaish et al., 2009). Behavioral aspects, such as delay of gratification, may allow for prioritizing others’ well-being and long-term benefits over immediate self-interest. Overall, a comprehensive theory emerges from these models, indicating that a combination of cognitive, affective, and behavioral regulatory processes provides a strong foundation for understanding and promoting PB. Differences in relation patterns between PB and single SR competences could become more pronounced when taking into account different PB trajectories and when considering different SR competences simultaneously. A more fine-grained understanding of potential differential links between trajectories and patterns of SR competences could be used to identify children in need for intervention early on and to derive tailored intervention measures targeting the lack of specific competences for different trajectories. For example, fostering emotion regulation generally improved PB (Schütz and Koglin, 2022, for an overview), but other competences may also be relevant or be even more relevant for children on specific PB trajectories.

### The present study

The present study aimed to address these gaps in research by identifying PB trajectories in a large sample of German children between 6 and 13 years of age over the course of 3 years and by relating the resulting trajectories to comprehensive patterns of numerous SR competences from three measurement points in middle childhood and into early adolescence, covering a highly relevant age range and using a multi-informant approach with experimental child measures as well as parent- and teacher reports. A multi-informant approach was used in order to not overstrain single raters when assessing multiple SR competences and because parents and teachers are differentially suited to assess some of them: For example, parents may be better qualified to assess children’s emotional reactivity and emotion regulation because they can observe their children also in private contexts while teachers may be better qualified to assess children’s planning behavior, because they can use their experiences with many children as a reference (Rescorla et al., 2014). Using a multi-informant, multi-method approach, therefore, allows to assess multiple SR competences. The study also aimed to add to existing research on individual differences in PB development in this age range as well as on SR competences as potential predictors of these developmental differences. From the theoretical considerations and empirical findings outlined above, we derived the following hypotheses: First, we expected to find positive relations between PB and the SR competences inhibition, updating, flexibility, emotion regulation, planning behavior, and delay of gratification as well as negative correlations between PB and emotional reactivity (Hypothesis 1). Second, integrating the outcomes of previous research on PB trajectories, we expected to find trajectories of stable low, stable moderate, stable high, and increasing PB (Hypothesis 2a) with most participants belonging to the stable moderate and fewest participants belonging to the stable low trajectory (Hypothesis 2b). Third, should we be able to find these trajectories, we expected children on the stable high PB trajectory to show particularly high inhibition, updating, flexibility, emotion regulation, planning behavior, and delay of gratification as well as low emotional reactivity at T1 (Hypothesis 3a); children on the stable low PB trajectory to show particularly low inhibition, updating, flexibility, emotion regulation, planning behavior, and delay of gratification as well as high emotional reactivity at T1 (Hypothesis 3b); children on the increasing PB trajectory to show low emotion regulation, high emotional reactivity, and average to high inhibition, updating, and flexibility at T1 (Hypothesis 3c); children on the stable moderate PB trajectory to show average to high inhibition, updating, flexibility, emotion regulation, planning behavior, and delay of gratification as well as low emotional reactivity at T1 (Hypothesis 3d). We explored other potential differences between trajectories in the further SR competences in our study as well as the SR competences at T2 and T3.

## Materials and methods

### Participants and procedure

We used data from 1,657 children and adolescents who participated in the PIER-study on intrapersonal developmental risks. Data collection took place in 2012 (T1), 2013 (T2), and 2015 (T3). At T1, participants were aged 6–11 years (*M =* 8.36, *SD =* 0.93, 52% female), at T2 they were aged 7–11 years (*N =* 1,612, *M =* 9.12, *SD =* 0.93, 52% female), and at T3 they were aged 9–13 years (*N =* 1,501, *M =* 11.07, *SD =* 0.92, 52% female). The parent’s highest degree of education was used as an indicator of the socio-economic status (SES). Response options ranged from *no degree* (1) to *university degree* (6). When data of both parents was available, we used the highest degree; when data of only one parent was available, we used this degree (no degree or support school diploma = 1%, lower secondary school diploma = 2%, secondary school diploma = 25%, A levels = 17%, university degree = 41%). For the parent-reports, we do not have any data on which parent (mother or father) filled out the questionnaire. At T1, *N* = 1,261 parents and *N* = 1,202 teachers; at T2, *N* = 1,137 parents and *N* = 990 teachers; and at T3, *N* = 994 parents and *N* = 973 teachers filled in the respective questionnaire. These two rater-sources will be combined into “other-reports” in the analysis.

The sample was recruited from 33 public primary schools in the Federal State of Brandenburg, Germany. The researchers asked the head of schools to participate, then children as well as their parents and teachers were invited to participate. Children received candy and little presents as well as a cinema (T1, T2) or bookstore (T3) voucher worth 5€ for their participation. Teachers received 5€ for the class fund for each child that participated in the study and for which they filled in the teacher questionnaire. Children’s data were collected in two sessions at school, home, or the university. Each session lasted about 45–60 min. Children were tested alone. Trained research assistants conducted the standardized examination procedures and read out instructions and items. Parent and teacher data were collected via online and paper-pencil questionnaires, filling out separate questionnaires for each child. Participation was voluntary. Children gave verbal assent and primary caregivers provided informed written consent. The Ethics Committee at the University of Potsdam, approved of all questionnaires and proceedings. We preregistered the variables, hypotheses, and analyses for this study: https://osf.io/jsgzy/?view_only=a0e24c97c1a9425c9c2aaee0ae775362 (see Appendix for further details).

### Measures

#### Prosocial behavior

Parents answered the five items (e.g., “likes to share with other children”) from the “prosocial-behavior” subscale of the Strengths and Difficulties Questionnaire (SDQ; Goodman, 1997) at T1, T2, and T3, respectively. Response options were (0) *not true*, (1) *somewhat true*, or (2) *definitely true*. The questionnaire has been shown to be reliable and valid, with Cronbach’s *α* = 0.64 for the parent-reported subscale “prosocial-behavior” (Goodman, 2001). We computed a total score by adding the responses to all five items.

#### Inhibition

Parents answered 6 items of the subscale “inhibitory control” (e.g., “my child can stop when told to stop”) of the Temperament in Middle Childhood Questionnaire (TMCQ; Simonds, 2006) at T1, T2, and T3. Response options ranged from (1) *does not apply* to (5) *applies*. The questionnaire has been shown to be reliable and valid, with Cronbach’s *α* = 0.80 for the parent-reported subscale “inhibitory control” (Simonds, 2006). We computed a mean score. Higher scores indicate higher SR.

#### Working-memory updating

We measured updating at T1, T2, and T3 using the “Digit Span Backward” task from the HAWIK-IV test of intelligence (Petermann and Petermann, 2010). Studies support the validity and reliability of the HAWIK-IV (Daseking et al., 2010; Hagmann-von Arx et al., 2012). Children were instructed to repeat digits from 0 to 9 in the reverse order in which they had been presented. Test leaders presented one digit per second with constant emphasis. There was one sample trial followed by up to eight core trials with an increasing number of digits. Each core trial consisted of two sequences. During the first trial, two digits had to be repeated in reverse order, then three, then four, and so on. During each trial, at least one of the two-digit sequences had to be reversely repeated correctly in order to proceed to the next trial. The dependent variable was the total number of correct trials and ranged between 0 and 16. Higher scores indicate higher SR.

#### Flexibility

We assessed flexibility at T1 and T2 with the Cognitive Attention Shifting Task (Roebers and Kauer, 2009; Röthlisberger et al., 2010). Children saw a single-colored and a multi-colored fish on the left- and right-handed side of a computer screen in 46 trials with interstimulus intervals of 300–700 ms. Children were instructed to alternatingly feed the single- and the multi-colored fish by pressing the corresponding keys on a QWERTZ-keyboard (“X” for the left side and “M” for the right side fish). The side on which the fish appeared switched between some trials (“switch trials”). Thus, children had to remember their previous response and to change their response pattern in switch trials (e.g., from pressing left/right to left/left). The number of correct responses in the 22 switch trials served as dependent variable. The Cognitive Attention Shifting Task has been shown to be valid and reliable measures of flexibility, with a split-half reliability of *r* = 0.70 (Roebers and Kauer, 2009; Röthlisberger et al., 2010). At T3, this task was replaced with an age appropriate task, an adapted version of the dimensional change card sort task (Qu et al., 2013). For this computerized task, children had to match symbols according to color or shape in 68 trials. Children saw two symbols in different color and shape at the top of the screen and one symbol below. Children were supposed to match the lower picture with one of the upper pictures by pressing the corresponding keys on a QWERTZ-keyboard (“F” for the upper left image and the “J” for the upper right image) depending on the sorting rule presented in the middle of the screen (color or shape). For example, if the symbol in the middle was a blue triangle and the sorting rule was “color” the children had to match it with the upper picture that was blue as well, independent of its shape. Children were instructed to react as quickly as possible and to make as few mistakes as possible. The number of correct responses for the 12 switch trials served as the dependent variable. Higher scores indicate higher flexibility and SR.

#### Emotional reactivity

Parents answered 10 items of the subscale “emotional control” (e.g., “overreacts to minor problems”) of the Behavior Rating Inventory of Executive Function (BRIEF; Gioia et al., 2000) at T1, T2, and T3, as well as 7 items of the subscale “anger/frustration” (e.g., “my child gets angry when it can‘t find something it’s looking for”) from the TMCQ (Simonds, 2006) at T1 and T2. Response options ranged from (1) *never* to (5) *always* for the BRIEF and from (1) *totally disagree* to (5) *totally agree* for the TMCQ. Both measures have been shown to be reliable and valid, with Cronbach’s *α* ranging from 0.80 to 0.98 for the BRIEF (Gioia et al., 2000) and Cronbach’s α = 0.88 for the parent-reported subscale “anger/frustration” of the TMCQ (Simonds, 2006). For T1 and T2, we calculated the total emotional-reactivity score by averaging the mean of both subscales. The subscales showed high correlations at T1 (*r* = 0.62) and T2 (*r* = 0.62). At T3, emotional reactivity was only indicated by the mean score of the emotional-control subscale. Emotional-reactivity scores correlated at *r* = 0.75 between T1 and T2, at *r* = 0.58 between T1 and T3, at *r* = 0.61 between T2 and T3, suggesting strong overlaps between measurement points despite changes in measurement. Higher scores indicate lower SR, respectively.

#### Emotion regulation

Parents rated 6 adapted items of the subscale “dealing with anger” from the Questionnaire to Assess Emotion Regulation in Children and Youths (FEEL-KJ; Grob and Smolenski, 2005) at T1 and T2. Response options ranged from (1) *never* to (5) *always*. The items measured four emotion-regulation strategies: Distraction (1 item), preservation (1 item), venting (2 items), and control of emotion (2 items). Based on the results of a Confirmatory Factor Analysis (CFA) using T1 data, we excluded the two items measuring control of emotion due to low factor loadings. At T3, venting was measured by one item only. We calculated the emotion-regulation total score by taking the mean of the 4 items at T1 and T2 and by taking the mean of the three items at T3. Emotion-regulation scores correlated at *r* = 0.48 between T1 and T2, at *r* = 0.43 between T1 and T3, and at *r* = 0.45 between T2 and T3, suggesting similar overlaps between measurement points despite changes in measurement. Scores were recoded so that higher scores indicate better emotion regulation and SR. The FEEL-KJ has been shown to be reliable and valid measure with Cronbach’s *α* between 0.82 and 0.93 (Cracco et al., 2015).

#### Affective decision-making

We measured affective decision-making at T1, T2 and T3 using age-adapted versions of the Hungry Donkey Task (Crone and van der Molen, 2004). Children were instructed to help the hungry donkey by collecting as many apples as possible in 60 trials. In each trial, children saw four closed doors on a computer screen and had to choose one of these doors by pressing the corresponding key on a QWERTZ keyboard (“S” for the outer left, “D” for the middle left, “K” for the middle right, “L” for the outer right door). By pressing one of the keys, the corresponding door opened, revealing the number of won (green) and lost (red) apples. The total net sum across trials was presented below the outer right door. The doors differed in their underlying win/loss contingencies: The doors on the left generated higher wins in single trials, but were disadvantageous in the long run, because they also generated higher losses across trials. Doors on the right generated lower wins in single trials, but were advantageous in the long run, because they generated lower losses across trials. The net score of advantageous/disadvantageous choices of the last 50 trials served as dependent variable [(K + L)-(S + D)], because win/loss contingencies are not yet obvious during the first trials (Brand et al., 2007). Higher scores represented less affective decision-making and better SR.

#### Planning behavior

Teachers answered 8 items (e.g., “Does not plan tasks for school in advance”) of the subscale “plan/organize” of the BRIEF (Gioia et al., 2000) at T1, T2, and T3. Response options ranged from (1) *never* to (5) *always*. The BRIEF has been shown to be a reliable measure (Gioia et al., 2000). We computed the total score by taking the mean of the 8 items. Scores were recoded so that higher scores indicate better SR.

#### Delay of gratification

To assess delay of gratification at T1, T2, and T3, we used four decision making trials including real incentives. Children were asked whether they would prefer a smaller gift now or a larger gift about 1 week later at the second test session (Wulfert et al., 2002). At T1 and T2, two food (chocolate, chewing candy) and two non-food (sticky tattoo, leap frog) stimuli were presented, respectively. Because the non-food stimuli should no longer be of immediate attraction, four food stimuli were presented at T3 (gummi bears, chewing gums, sherbet powder, chocolate bar). The smaller reward was shown to the children. The larger reward was a higher quantity of the smaller reward (e.g., “one chewing candy now or a pack of chewing candies in a week”). We asked children whether or not they were allowed to eat sweets or were intolerant to the sweets prior to the offer. If so, children were asked to answer the questions as though they were able to eat the sweets. Additionally, children were asked whether or not they really wanted to receive the items. The dependent variable was the sum of the delayed items. Higher scores represented higher delay of gratification and better SR. This measure has been shown to have good convergent validity (Pollatos et al., 2023).

### Analysis

We computed mean values, standard deviations, internal consistencies, Multivariate Analyses of Covariance (MANCOVA), as well as zero-order correlations to test Hypothesis 1 for all measures using IBM SPSS Version 28. We also calculated effect sizes whereby η^2^_p_ ≤ 0.01 reflected a small effect size, η^2^_p_ ≤ 0.06 a medium effect size, and η^2^_p_ > 0.14 a large effect size. To test the second hypothesis on trajectories of PB, we computed Latent Class Growth Analyses (LCGA) including PB across all three measurement points using M*plus* Version 8.6 (Muthén and Muthén, 1998-2017). The dropout rate was small (2.5% from T1 to T2, 6.7% from T2 to T3). We accounted for missing data by using the full information maximum likelihood (FIML) procedure that allowed for the retention of the full T1/T2/T3 sample across measurement points. We tested LCGA models with one class up to five classes. We used the Akaike information criterion (AIC), the Bayesian information criterion (BIC), the Vuong-Lo–Mendell–Rubin likelihood ratio test (VLMR), entropy, and the lowest class size to determine the best fitting model. Lower AIC and BIC values indicate a better model fit. The VLMR test examines whether the current model fits the data better than the model with one class less (Nylund et al., 2007). A model with a significant VLMR test, thus, fits the model better than the model with one class less. To ensure that the PB measure had the same meaning across measurement points, we tested configural, weak, strong, and strict measurement invariance (MI). The results indicated strong measurement invariance when considering fit indices that are not sensitive to sample size (AIC, BIC, CFI, RMSEA, and SRMR; Table 1). Thus, changes in PB in the present study likely can be attributed to changes in the frequency of this behavior. To test for differences in PB levels between the four trajectories, we conducted a MANOVA accounting for multiple comparisons using the Bonferroni-Holm correction.

**Table 1:** Test for measurement invariance of prosocial behavior for the three measurement points.

	Configural	Weak	Strong	Strict
AIC	22,037.829	22,026.733	22,024.029	22,040.211
BIC	22,371.160	22,317.737	22,272.705	22,235.977
RMSEA	0.016	0.014	0.015	0.020
CFI	0.993	0.993	0.992	0.985
SRMR	0.022	0.024	0.025	0.049
χ*^2^*	98.93	103.83	117.13	153.31
Δχ*^2^*	–	4.905	13.296	36.181
Δχ*^2^ p*-value	–	0.768	0.102	0.000

AIC, Akaike’s information criterion; BIC, Bayesian information criterion; RMSEA, root mean square error of approximation; CFI, comparative fit index; SRMR, root mean square residual; Δχ^2^, difference in χ^2^ toward the less restrictive model.

**Figure 1:**
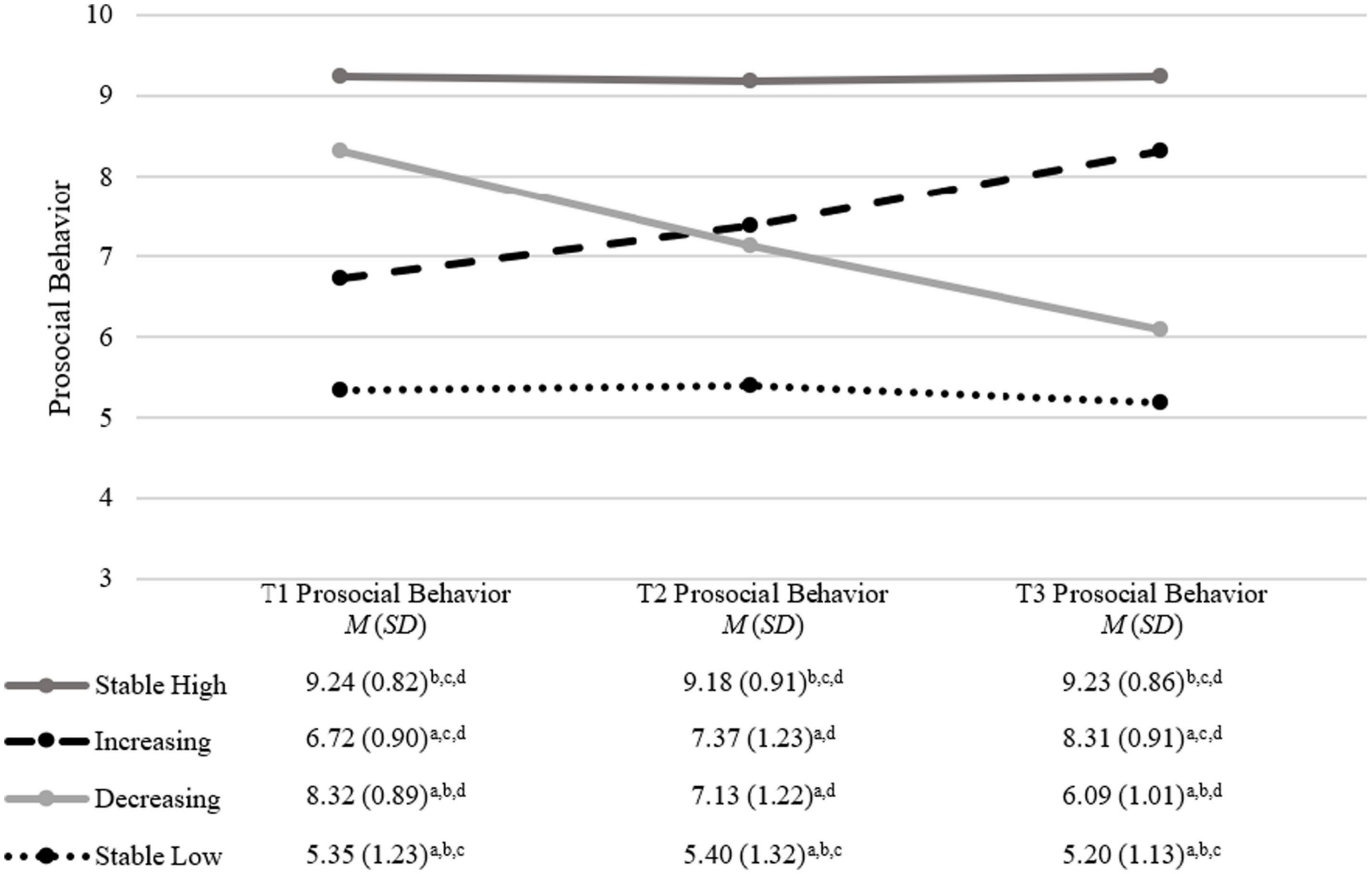
Prosocial-behavior trajectories from middle childhood to early adolescence. The figure illustrates distinct trajectories of prosocial behavior observed across middle childhood to early adolescence. The trajectories are categorized into: stable high, increasing, decreasing, and stable low; Statistical significance is indicated by the following letters: ^a^significantly differs from the stable high trajectory, ^b^significantly differs from the increasing trajectory, ^c^significantly differs from the decreasing trajectory, ^d^significantly differs from the stable low trajectory; adjusted via Bonferroni-Holm correction.

To test the third hypotheses on differences in SR competences between children on different PB trajectories, we computed one MANOVA including age, gender, and SES at T1 and six MANOVAs including the SR competences separated by measurement point and rater source (experimental: updating, flexibility, affective decision making, delay of gratification; other-report: inhibition, emotional reactivity, emotion regulation, planning behavior). To make the SR competences comparable in size and to visualize differences, we computed *z*-standardized mean values per measurement point (Figure 1). We considered experimental child measures and other-ratings separately in the MANOVAs for two reasons: First, this allowed us to retain a larger sample size and higher statistical power because the sample size for the other-ratings were significantly smaller than for the self-ratings, especially at T2 and T3 (T1: *N*_self_ = 1,131 and *N*_other_ = 978; T2: *N*_self_ = 1,137 and *N*_other_ = 689; T3: *N*_self_ = 1,121 and *N*_other_ = 633). Second, since we assumed to find both rater- and method effects, we wanted to prevent any confounding effects by analyzing both effects seperatly. We applied the Bonferroni-Holm correction to account for multiple comparisons.

## Results

### Descriptive statistics and correlations

Table 2 presents descriptive statistics and gender differences for all variables. Controlling for age and SES, MANCOVAs showed a main effect of gender at T1 [*F*(9, 873) = 15.24; *p* < 0.001; η^2^*_p_ =* 0.14], T2 [*F*(9, 627) = 10.15; *p* < 0.001; η^2^*_p_ =* 0.13], and T3 [*F*(9, 595) = 8.99; *p* < 0.001; η^2^*_p_ =* 0.12]. On subscale level, girls were rated to show higher prosocial behavior, inhibition, and planning behavior as well as lower affective decision making than boys at all measurement points, and higher flexibility at T1 and T2.

**Table 2:** Internal consistencies, mean values, standard deviations, and gender differences of all measures.

T1	*n*	α	Total *M* (*SD*)	Girls *M* (*SD*)	Boys *M* (*SD*)	Range	*F*	η^2^*_p_*
Prosocial behavior (o)	1,306	0.64	8.29 (1.60)	8.51 (1.52)	8.04 (1,65)	1–10	25.02*	0.025
Inhibition (o)	1,306	0.68	3.53 (0.66)	3.62 (0.63)	3.45 (0.68)	1.17–5	23.44*	0.024
Working-memory updating (e)	1,443	–	6.23 (1.46)	6.21 (1.47)	6.24 (1.46)	0–13	0.10	0.000
Flexibility (e)	1,445	–	15.72 (4.59)	16.48 (4.51)	14.90 (4.53)	0–22	46.74*	0.040
Emotional reactivity (o)	1,330	0.91	3.58 (0.66)	3.61 (0.64)	3.54 (0.68)	1.47–5	2.89	0.003
Emotion regulation (o)	1,297	0.55	2.19 (0.66)	2.23 (0.65)	2.14 (0.66)	1–5	2.40	0.002
Affective decision making (e)	1,445	–	5.44 (11.40)	4.06 (9.62)	14.90 (4.53)	−32-50	19.39*	0.017
Planning behavior (o)	1,202	0.93	3.76 (0.89)	3.95 (0.83)	3.55 (0.90)	1–5	51.68*	0.050
Delay of gratification (e)	1,306	0.56	2.81 (1.4)	2.73 (1.23)	3.31 (0.99)	0–4	5.54	0.005
T2								
Prosocial behavior (o)	1,182	0.65	8.25 (1.63)	8.55 (1.49)	7.93 (1.72)	1–10	31.13*	0.043
Inhibition (o)	1,174	0.67	3.59 (0.63)	3.66 (0.62)	3.50 (0.64)	1.17–5	12.84*	0.018
Working-memory updating (e)	1,426	-	6.67 (1.05)	6.68 (1.49)	6.65 (1.52)	0–13	1.25	0.001
Flexibility (e)	1,408	-	18.29 (3.77)	18.72 (3.66)	17.83 (3.84)	0–22	20.50*	0.018
Emotional reactivity (o)	1,192	0.91	3.64 (0.63)	3.65 (0.61)	3.63 (0.66)	1–5	0.73	0.001
Emotion regulation (o)	1,164	0.50	2.24 (0.63)	2.52 (0.64)	2.22 (0.62)	1–5	0.00	0.000
Affective decision making (e)	1,408	-	8.52 (12.83)	6.75 (11.00)	6.65 (1.52)	−32-50	17.18*	0.015
Planning behavior (o)	990	0.93	3.69 (0.89)	3.91 (0.82)	3.45 (0.90)	1–5	55.24*	0.075
Delay of gratification (e)	1,357	0.51	3.29 (0.96)	3.27 (0.93)	2.90 (1.24)	0–4	0.68	0.001
T3								
Prosocial behavior (o)	1,054	0.69	8.34 (1.65)	8.62 (1.57)	8.04 (1.69)	2–10	12.73*	0.020
Inhibition (o)	1,055	0.71	3.57 (0.68)	3.82 (0.67)	3.68 (0.68)	1.33–5	8.17*	0.013
Working-memory updating (e)	1,351	-	7.39 (1.61)	7.41 (1.60)	7.37 (0.61)	1–13	0.27	0.000
Flexibility (e)	1,338	-	9.78 (1.86)	9.87 (1.91)	9.69 (1.81)	0–12	3.52	0.003
Emotional reactivity (o)	1,065	0.92	3.75 (0.73)	3.72 (0.74)	3.79 (0.72)	1.2–5	1.64	0.003
Emotion regulation (o)	1,048	0.35	2.31 (0.66)	2.33 (0.67)	2.29 (0.64)	1–4.33	0.68	0.001
Affective decision making (e)	1,339	-	9.46 (13.66)	7.63 (12.23)	11.48 (14.82)	−22-50	30.06*	0.026
Planning behavior (o)	973	0.94	3.74 (0.94)	3.91 (0.92)	3.57 (0.92)	1–5	23.84*	0.037
Delay of gratification (e)	1,350	0.65	2.29 (1.38)	2.22 (1.35)	2.36 (1.40)	0–4	3.06	0.003

^*^Significant after Bonferroni-Holm correction; (o), other-rated; (e), experimental data.

Table 3 presents the zero-order correlations between PB and the SR competences at T1, T2, and T3. There was a high stability of PB across measurement points ranging from *r = 0*.506 to 0.588. Partially supporting Hypothesis 1, T1, T2, and T3 inhibition and planning behavior showed consistent positive, and emotional reactivity showed consistent negative relations with PB at T1, T2, and T3. Furthermore, there were positive correlations between emotion regulation and PB at different measurement points and between T3 flexibility and T1 prosocial behavior. Expectedly, there were no relations with affective decision-making. In contrast to Hypothesis 1, there were no relations with updating or delay of gratification.

**Table 3:** Correlations between prosocial behavior and self-regulatory competences for the total group.

	1	2	3	4	5	6	7	8	9	10	11	12
1 T1 prosocial behavior												
2 T2 prosocial behavior	0.588^*^											
3 T3 prosocial behavior	0.506^*^	0.573^*^										
4 T1 age	0.028	−0.020	−0.008									
5 T1 SES	0.008	−0.002	0.019	−0.069^*^								
T1												
6 pInh	0.301^*^	0.279^*^	0.292^*^	0.050	0.127^*^							
7 WMU	0.011	−0.032	−0.020	0.250^*^	0.164^*^	0.095^*^						
8 Flx	0.048	0.022	−0.023	0.305^*^	0.171^*^	0.044^*^	0.352^*^					
9 pEmR	−0.311^*^	−0.261^*^	−0.219^*^	0.000	−0.024	−0.473^*^	−0.089^*^	−0.080^*^				
10 pEmReg	0.081^*^	0.058	0.076^*^	0.020	0.134^*^	0.134^*^	−0.013	−0.010	−0.389^*^			
11 ADM	−0.040	−0.018	−0.057	0.071^*^	0.051	0.018	0.076^*^	0.094^*^	−0.030	−0.037		
12 tPln	0.138^*^	0.128^*^	0.162^*^	0.055	0.244^*^	0.294^*^	0.255^*^	0.288^*^	−0.158^*^	−0.012	0.023	
13 DoG	−0.002	−0.011	−0.022	0.122^*^	0.025	0.034	0.056^*^	0.093^*^	−0.027	−0.018	0.029	0.112
T2												
6 pInh	0.247^*^	0.298^*^	0.288^*^	0.049	0.123^*^							
7 WMU	0.018	−0.008	−0.002	0.200^*^	0.128^*^	0.092^*^						
8 Flx	0.033	0.043	0.003	0.256^*^	0.168^*^	0.139^*^	0.319^*^					
9 pEmR	−0.261^*^	−0.305^*^	−0.252^*^	−0.033	−0.031	−0.461^*^	−0.088^*^	−0.092^*^				
10 pEmReg	0.66^*^	0.048	0.046	0.097^*^	−0.121^*^	0.100^*^	−0.014	−0.033	−0.366^*^			
11 ADM	0.013	0.011	−0.046	0.080^*^	0.100^*^	0.029	0.087^*^	0.116^*^	0.004	−0.002		
12 tPln	0.171^*^	0.152^*^	0.198^*^	0.021	0.226^*^	0.240^*^	0.265^*^	0.288^*^	−0.156^*^	−0.018	0.055	
13 DoG	−0.016	−0.012	−0.052	−0.004	0.010	−0.066^*^	0.017	0.017	0.024	−0.058	0.052	−0.015
T3												
6 pInh	0.217^*^	0.249^*^	0.346^*^	0.035	0.157^*^							
7 WMU	−0.009	−0.025	0.005	0.107^*^	0.126^*^	0.069^*^						
8 Flx	0.061^*^	0.030	−0.014	0.025	0.091^*^	0.096^*^	0.131^*^					
9 pEmR	−0.201^*^	−0.204^*^	−0.305^*^	0.006	−0.019	−0.338^*^	−0.061^*^	−0.007				
10 pEmReg	0.074^*^	0.047	0.131^*^	0.085^*^	−0.058	0.140^*^	0.020	0.013	−0.408^*^			
11 ADM	0.002	0.000	−0.011	0.055^*^	0.051	0.017	0.081^*^	0.024	0.002	−0.005		
12 tPln	0.089^*^	0.116^*^	0.165^*^	−0.091^*^	0.224^*^	0.278^*^	0.155^*^	0.145^*^	−0.211^*^	0.079^*^	0.047	
13 DoG	−0.048	−0.024	−0.048	−0.047	−0.003	0.035	−0.038	0.060^*^	0.029	−0.018	0.040	0.010

pInh, parent-report inhibition; WMU, working-memory updating; Flx, flexibility; pEmR, parent-report emotional reactivity; pEmReg, parent-report emotional regulation; ADM, affective decision making; tPln, teacher-report planning behavior; DoG, delay of gratification; ^*^significant after Bonferroni-Holm correction.

### Trajectory analysis

Table 4 shows the model fits of the 1- to 5-trajectory solutions of the LCGA models for prosocial behavior. AIC, BIC, LMR, and VLMR all indicated the best fit with the data of the 4-trajectory model. Based on these empirical results, the comparison of the smallest trajectory per model, as well as theoretical considerations (i.e., the 4-trajectory model often strikes a balance between complexity and parsimony, capturing sufficient variability in developmental paths), we chose this model although entropy was higher for the 3-trajectory (reflecting stable low, medium, and high) and 5-trajectory (reflecting stable low, stable high, low increasing, medium increasing, and decreasing) models. Partially supporting Hypothesis 2a, the trajectories reflected stable high, increasing, decreasing (instead of the expected moderate), and stable low levels of PB over the 3-year period. Of the total sample (*N* = 1,461), most participants belonged to the stable high trajectory (62%), followed by the increasing (20%), the decreasing (10%), and the stable low trajectory (9%), only partly supporting Hypothesis 2b (least participants belonged to the stable low, but most to the stable high instead of the expected moderate trajectory). Figure 1 shows the four trajectories and their mean values of PB across all measurement points. A MANOVA showed a main effect of trajectory membership on PB across all three measurement points [*F*(9, 2,083.43) = 343.97; *p* < 0.001; η^2^*_p_ =* 0.52]. Bonferroni-Holm corrected Games-Howell post-hoc tests showed that the PB mean scores differed between all trajectories at all measurement points (*p* < 0.001), except for the increasing and decreasing trajectory at T2.

**Table 4:** Trajectory model fits.

	AIC	BIC	Entropy	VLMR	LMR	% of participants in the smallest trajectory
1-trajectory model	13,508.611	13,535.056	–	–	–	–
2-trajectory model	12,523.204	12,565.499	0.794	0.000	0.000	26
3-trajectory model	12,370.757	12,428.913	0.732	0.031	0.035	8
4-trajectory model	12,276.297	12,350.314	0.720	0.045	0.050	9
5-trajectory model	12,190.295	12,280.172	0.764	0.105	0.112	1

AIC, Akaike’s information criterion; BIC, Bayesian information criterion; VLMR, Vuong-Lo–Mendell–Rubin; LMR, Lo–Mendell–Rubin.

### Differences between trajectories in SR competences and demographics

The results of the MANOVA including the demographic variables indicated a main effect of the trajectory [*F*(9, 3,005.82) = 4.42; *p = 0*.001; η^2^*_p_* = 0.01]. On a subscale level, there were differences in gender [*F*(3, 1,240) = 10.72; *p* < 0.001; η^2^*_p_* = 0.03], but not in T1 age or T1 SES. More specifically, 58% (*n* = 522) of the children on the stable high, 49% (*n* = 71) of the children on the decreasing, 44% (*n* = 128) of the children on the increasing, and 35% (*n* = 43) of the children on the stable low trajectory were girls.

**Figure 2:**
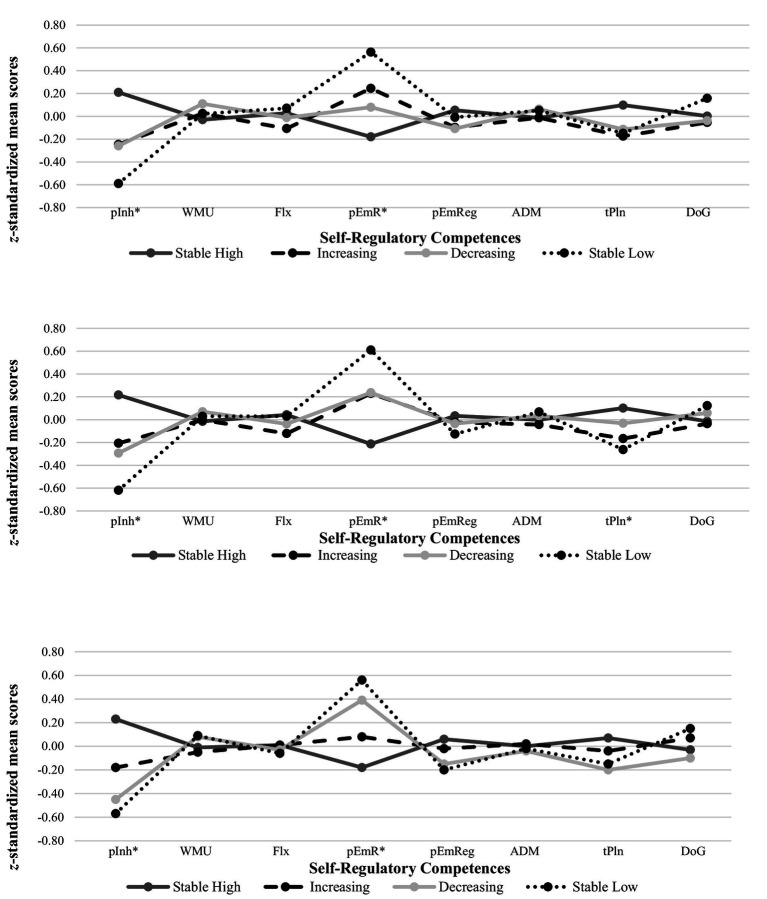
T1 patterns of self-regulation competences per each of the four prosocial-behavior trajectories. T2 patterns of self-regulation competences per each of the four prosocial-behavior trajectories. T3 patterns of self-regulation competences per each of the four prosocial-behavior trajectories. This figure displays the patterns of the self-regulation competences at the three measurement points (T1, T2, T3) categorized by the four different developmental trajectories in the present study; pInh, parent-report inhibition; WMU, working-memory updating; Flx, flexibility; pEmR, parent-report emotional reactivity; pEmReg, parent-report emotional regulation; ADM, affective decision making; tPln, teacher-report planning behavior; DoG, delay of gratification; *sign. Differences between at least two of the trajectories (adjusted via Bonferroni-Holm correction) (Table 5 for details).

Table 5 presents the mean values and Figure 6 the patterns of all SR competences across all measurement points per trajectory. In contrast to Hypotheses 3a to d, after adjusting via Bonferroni-Holm correction results of the MANOVAs including the experimentally measured SR competences did not show main effects of the trajectory at T1 [*F*(12, 3,495.33) = 1.57; *p = 0*.093; η^2^*_p_ = 0*.01]. Neither did the results show any main effects of the trajectory at T2 [*F*(12, 3,503.27) = 1.30; *p = 0*.210; η^2^*_p_* = 0.00] or T3 [*F*(12, 3,500.62) = 0.88; *p = 0*.566; η^2^*_p_* = 0.00]. Results of the MANOVAs including the other-reported SR competences showed main effects of the trajectory at T1 [*F*(12, 2,794.21) = 8.54; *p* < 0.001; η^2^*_p_ = 0*.03], T2 [*F*(12, 2,093.08) = 8.60; *p* < 0.001; η^2^*_p_ = 0*.04], and T3 [*F*(12, 1,852.32) = 8.64; *p* < 0.001; η^2^*_p_ =* 0.05]. On subscale level, there were significant overall differences between trajectories for inhibition and emotional reactivity at all three measurement points and for T2 planning behavior. Follow-up analyses with Games-Howell post-hoc tests showed that partially supporting Hypothesis 3a, children on the stable high trajectory showed higher inhibition and planning behavior, as well as lower emotional reactivity than children on the other trajectories (see Table 5 for a more detailed differentiation per measurement point and trajectory). Partially supporting Hypothesis 3b, children on the stable low PB trajectory not only differed from children on the stable high trajetory, but also showed higher T1 and T2 emotional reactivity than children on the decreasing trajectory. There were no differences in the SR competences between children on the increasing and decreasing PB trajectory, contrasting Hypothesis 3c. In addition, inspections of the SR patterns (Figure 2) suggested decreases in inhibition and planning as well as increases in emotional reactivity for children on the decreasing trajectory from T1 and T2 to T3. Opposite patterns were found for children on the increasing trajectory. Exploratory follow-up analyses, however, showed no significant differences. Because there was no stable moderate trajectory, Hypothesis 3d was irrelevant.

**Table 5:** Z-standardized means of self-regulation competences separated by trajectories and measurement points.

		pInh	WMU	Flx	pEmR	pEmReg	ADM	tPln	DoG
Stable high	T1	0.21^b,c,d^	−0.03	0.03	−0.18^b,d^	0.05	−0.01	0.10	0.00
	T2	0.22^b,c,d^	−0.01	0.04	−0.21^b,d^	0.03	0.00	0.10^d^	−0.01
	T3	0.23^b,c,d^	−0.01	0.01	−0.18^c,d^	0.06	0.00	0.07	−0.03
Increasing	T1	−0.25^a^	0.03	−0.11	0.25^a^	−0.10	−0.01	−0.17	−0.05
	T2	−0.21^a^	0.00	−0.12	0.23^a^	−0.02	−0.04	−0.17	−0.04
	T3	−0.18^a^	−0.05	0.01	0.08	−0.02	0.02	−0.04	0.07
Decreasing	T1	−0.26^a^	0.11	−0.01	0.08^d^	−0.11	0.06	−0.11	−0.04
	T2	−0.29^a^	0.07	−0.04	0.24^d^	−0.03	0.03	−0.03	0.06
	T3	−0.45^a^	0.08	−0.03	0.39^a^	−0.15	−0.04	−0.20	−0.10
Stable low	T1	−0.59^a^	0.02	0.07	0.56^a,c^	−0.01	0.05	−0.15	0.16
	T2	−0.62^a^	0.03	0.03	0.61^a,c^	−0.13	0.07	−0.26^a^	0.12
	T3	−0.57^a^	0.09	−0.06	0.56^a^	−0.20	−0.02	−0.15	0.15

pInh, parent-report inhibition; WMU, working-memory updating; Flx, flexibility; pEmR, parent-report emotional reactivity; pEmReg, parent-report emotional regulation; ADM, affective decision making; tPln, teacher-report planning behavior; DoG, delay of gratification; ^a^significantly differs from the stable high trajectory; ^b^significantly differs from the increasing trajectory; ^c^significantly differs from the decreasing trajectory; ^d^significantly differs from the stable low trajectory; adjusted via Bonferroni-Holm correction.

## Discussion

The present findings indicate that prosocial behavior (PB) requires effective self-regulation (SR). Yet, little is known about the potential differential relations between PB trajectories from middle childhood into early adolescence and patterns of multiple SR competences. Supporting previous research and our hypotheses, the present study showed consistent positive associations between most SR competences and PB in general. In line with our second hypothesis, however, we identified four distinct PB trajectories that reflected stable low and high as well as increasing and decreasing levels of PB over the three measurement points and the 3-year period. Finally, we found stable differences in SR competences between children on these four trajectories particularly in inhibition, emotional reactivity, and planning behavior, with most beneficial expressions among children on the stable high PB trajectory. Thus, children on different PB trajectories show early and continuous differences in SR competences. Assuming that SR can positively influence PB development, these findings allow for an early identification of children in need of intervention that should particularly address inhibition, emotional reactivity, and planning behavior.

### PB and SR competences

Positive correlations between general PB and inhibition, planning, as well as emotion regulation, negative correlations with emotional reactivity, and no correlations with affective decision-making support previous research (Benita et al., 2017; Carlo et al., 2012; Moriguchi et al., 2020; O’Toole et al., 2017; Schütz and Koglin, 2022; Steinbeis and Over, 2017), our theoretical expectations that PB requires effective SR, and underscore the reliability and validity of the present findings particularly regarding the other-reported SR competences in our study. However, no correlations between PB and the experimentally measured SR competences updating, flexibility, and delay of gratification contradicted both previous research (Moriguchi et al., 2020; Paulus et al., 2015; Traverso et al., 2020) and our expectations. The lack of associations between PB and all experimental SR measures in the present study may indicate that there are more important SR competences, but it may also reflect measurement issues: For example, there were ceiling effects and low reliabilities of the delay of gratification measure. In addition, rater effects may limit the overlaps between parent-rated PB and experimentally measured SR competences. Furthermore, we can not rule out the possibility that the associations found between parent-reported PB and parent reported inhibition are due to similarities in measurement scales, which both assess aspects of social desirability.

### PB trajectories

The identification of a stable high, increasing, decreasing, and stable low PB trajectory with most children on the stable high and least children on the stable low trajectory mainly corroborated previous research (Côté et al., 2002; Flynn et al., 2015; Griese et al., 2016; Lee et al., 2022; Shi et al., 2021) and our hypotheses. Contrasting our hypothesis, our findings underscore recent research that found increasing and decreasing trajectories rather than a stable moderate trajectory (Lee et al., 2022; Shi et al., 2021).

Our findings suggest that more than 70% of the children in our study showed stable high or stable low PB levels from middle childhood onwards, suggesting a high stability of PB from middle childhood and into adolescence. This also implies that roughly one third of the children in our sample showed changes in their PB levels during this three-year period, resulting in roughly 80% of the total sample that reached high levels of PB by the end of childhood and beginning of adolescence. However, almost two fifths of the children in our sample showed low levels of PB on at least two measurement occasions and roughly one fifth showed problematic PB at T3 (also reflecting the portion of children that can be expected to show problematic PB as suggested by SDQ cut-off score; Goodman, 1997). Interestingly, children on the decreasing and increasing trajectories never scored as high or as low in PB as children on the stable high and stable low trajectory, respectively. This makes the four trajectories distinguishable by their levels of PB alone and allows for distinguishing between children with and without potential need for intervention early on. Of note, however, some children may undergo PB development primarily during adolescence and/or in a cubic trend (Carlo et al., 2007; Luengo Kanacri et al., 2013). Such a cubic trend may even elucidate the observed decreasing and increasing trajectories: Early maturing children may already have traversed the increasing path of the cubic trend, whereas later maturing children may still be progressing along the decreasing path during middle childhood. Furthermore, some researchers argue that decreases in PB throughout middle childhood are related to children’s increased understanding of reciprocity leading to less indiscriminate altruism (Warneken and Tomasello, 2009). However, we argue that this explanation is unlikely in the context of our study due to the nature of our measurement method: PB was assessed through parent-reports, providing a specific perspective on their child’s behavior that may differ from direct observations or self-reports. Parent-reports capture how parents perceive their child’s behavior in various contexts over time and are, thus, influenced by general impressions and consistent behaviors rather than situational nuances (Achenbach, 1991).

### PB trajectories and SR competences

In line with our expectations, participants on the four PB trajectories also differed in their SR competences. Thus, the present study extends the existing knowledge about variables that are associated with differential PB development by focusing on internal factors that have rarely been considered by previous research and that are important because they may be addressed by child-focused intervention measures for example in the school context (Diamond and Lee, 2011). Specifically, results showed that trajectory membership was consistently associated with inhibition and emotional reactivity in all age groups and with planning behavior in middle childhood, suggesting that these SR competences are key skills for PB. Inhibition may be particularly important for PB development because it enables children to control their responses before acting and to ponder different response options and their likely short- and long-term consequences for themselves and others (Leshem et al., 2020). This is in line with past research that indicated that inhibition is a central feature for other SR competences, such as updating, flexibility, and planning behavior (Kor and Mullan, 2011). Our findings indicate that next to successfully suppressing primary impulses, children also need to think about the future and plan prosocial actions while taking into consideration what their goals are and how to achieve them (Baumsteiger, 2017; Leshem et al., 2020). Finally, low emotional reactivity may benefit PB, because children who frequently and intensely experience negative emotions, particularly anger, may be more inclined to show disfavorable social responses (Calkins et al., 1999). According to cognitive-load theory, the experience of strong negative emotions demands cognitive resources that interfere with cognitive abilities (e.g., inhibition) needed for PB (Gabel and McAuley, 2020).

The finding that these SR competences tended to distinguish children on the stable high PB trajectory from children on all other trajectories suggests to promote them in all children in terms of universal prevention or in all children that do not score high in PB in terms of selected prevention regardless of the specific PB trajectory. In line with previous research (Côté et al., 2002; Flynn et al., 2015; Malti et al., 2013), this may be particularly helpful in boys, who were less frequently on the stable high PB trajectory than girls, but more frequently on the increasing trajectory, suggesting a somewhat later PB development in boys (Van der Graaff et al., 2018). In addition, particularly children with decreasing PB levels may profit from interventions so that a spiral of negative outcomes may be prevented early on. A closer look at the developmental trends of the SR competences (see Table 5 and Figure 2) supports this assumption because findings suggest decreases in SR, especially in inhibition and planning behavior, and increases in emotional reactivity from T1 to T3 as well as the opposite pattern among children on the increasing trajectory. These trends suggest that changes in PB correspond to changes in SR. Furthermore, the findings suggest developmental differences between PB trajectories particularly for emotional reactivity, which only become apparent in early adolescence (i.e., children in the increasing trajectory show lower emotional reactivity and do not differ from children on the high stable trajectory for the first time at T3).

Future research should shed further light on the question which of the three SR competences that turned out to be relevant in the present study should primarily be addressed by intervention measures. On the one hand our results show that inhibition and emotional reactivity had the most reliable and strongest differences between trajectories, and particularly inhibition seems to be underlying other self-regulatory skills (Kor and Mullan, 2011; Miyake et al., 2000). On the other hand, emotional reactivity is often considered as a temperamental characteristic rather than a competence that, therefore, may be less open to change and influence than the other competences. Being a cognitively based process, planning behavior seems to be prone to individual intervention measures and should, thus, also be considered in future research (MacLeod et al., 2008).

### Limitations and outlook

The strengths of the present study include the large sample size, the longitudinal design, the consideration of multiple SR competences and rater sources, as well as relating patterns of SR competences that can be addressed by child-focused interventions to different PB trajectories. Limitations include the lack of a single underlying theoretical framework providing the rationale for the SR competences under examination, the reliance on parent-reports on PB that may be susceptible to response bias, as well as smaller response rates for other-rated than for experimentally measured SR variables. Thus, MANOVAs testing for differences in SR competences were separated by rater source and measurement point to maximize statistical power, which prevented us from simultaneously testing the effects of all competences. The results indicated measurement effects. Therefore, future research should assess all constructs by different methods and control for method effects in statistical analysis. Further, differences in SR between trajectories were mainly visible for children on the stable high trajectory and more difficult to detect between the stable low, increasing, and decreasing trajectories. This is most likely due to small group sizes of these trajectories, causing lower power. Additionally, the differences between trajectories were mainly visible in two parent-reported SR scales (inhibition and emotional reactivity). However, because there were also differences between children on the stable high and low trajectories in teacher-reported planning behavior, and because children on different trajectories did not show differences on all parent-reported SR, the significant associations are likely not merely due to rater effects. Internal consistencies were low for emotion regulation at all measurement points and for delay of gratification at T1 and T2. Future studies should, therefore, use other measurement methods for these constructs. Because we do not have data on children’s SR competences prior to the first measurement of PB, we cannot draw any causal inferences about their influence on the PB trajectories. A fourth measurement point would have been necessary to do so. Finally, changes in PB may be due to external influences that were beyond the scope of the present study. For example, this study did not control for the effects of school transitions and changes in peer groups, which can introduce new social dynamics that may impact PB differently across various ages. Such external influences should be considered by future research to control for their potential effects. Likewise, to increase the interpretability of results, future studies should include more confounding factors, such as social desirability, parenting behaviors, and peer influence.

The present findings add to the existing knowledge on PB development by relating differential trajectories to patterns of multiple SR competences across middle childhood. Thereby, the findings highlight our initial assumption on the importance of SR for PB. The majority of children in the present study showed stable high levels of PB, but a large minority of 38% showed low PB on at least two measurement occasions. Of note, children on these trajectories differed in their levels of PB as well as in their pattern of SR competences, particularly inhibition, emotional reactivity, and planning behavior early on and consistently. This allows for an early distinction between children with and without the need for interventions targeting these aforementioned SR competences in particular. Contrary to our expectations, differences in SR were mainly visible between children on the high trajectory and the other trajectories, indicating that children on the other trajectories (i.e., increasing, decreasing, stable low) can benefit from universal intervention programs. Additionally, a substantial minority of children showed low PB on at least two measurement occasions.

These findings underscore the importance of targeting these specific SR competences in clinical and educational settings as well as for parenting practices at home in order to promote PB. In clinical contexts, practitioners should focus particularly on the children’s abilities in inhibition and planning behavior, as well as their emotional reactivity when working with children who have problems with PB. In schools, SR-focused early screening tools could help educators identify children who may benefit from SR competence-building programs early on, promoting a positive development. Programs that encourage children to practice “stop-and-think” methods before responding (e.g., The Red Light, Purple Light Intervention; Tominey and McClelland, 2011) can be integrated into classroom routines to enhance inhibition, whereas social emotional learning can improve both inhibition and emotional reactivity through exercises in self-control, recognizing and managing feelings, and interpersonal conflict resolution (Diamond and Lee, 2011; Liew and Spinrad, 2022). Planning skills could be cultivated through exercises, building positive engagement with goals and organization skills (MacLeod et al., 2008). Additionally, teachers could be trained in behavioral observation techniques (to recognize SR deficits early on and provide timely support) and responsive classroom management (e.g., setting clear expectations, using positive reinforcement) to create SR-supportive environments. Such exercises are most effective when integrated daily (Jones and Bouffard, 2012). Furthermore, mindfulness practice has been shown to support inhibition and PB and to counter strong emotional reactivity (Flook et al., 2015). These interventions should be reinforced outside the classroom (Goldberg et al., 2019): For example, parents should model SR, maintaining consistent rules and rewards, and establishing supportive routines, creating a cohesive framework that encourages SR competences across home and school settings.

Next, to these practical implications, our findings underscore important empirical and theoretical implications: Empirically, the observed patterns provide new insights into developmental trajectories of PB in middle childhood. Theoretically, they highlight the complex interplay between SR and PB, suggesting that a multifaceted approach is needed to understand these relationships better. Future research should focus on corroborating these findings with a broader range of measures, including direct observations and self-reports of all measures, to ensure a comprehensive understanding of SR and PB development. By emphasizing the empirical and theoretical consequences of our findings, we aim to contribute to the broader discourse on promoting PB and SR in childhood development.

#### Public significance statement

This study shows that children differ in their prosocial-behavior development from middle childhood to early adolescence, with most children continuously showing high levels of prosocial behavior. Differences in prosocial-behavior development were associated with differences in children’s self-regulation, specifically inhibition, emotional reactivity, and planning behavior. The results suggest that these skills should be the focus of early interventions targeting all children who exhibit either lower prosocial behavior or self-regulation values to promote children’s prosocial-behavior development.

## Data Availability

The datasets presented in this article are not readily available because the raw data supporting the conclusions of this article are part of a larger longitudinal study with strict data protection guidelines. Upon completion of the ongoing data collection and publications, data will be made available in a public repository. Requests to access the datasets should be directed to Rebecca Bondü, r.bondue@phb.de.

## Appendix – deviations from the pre-registration

- We used the label “emotional reactivity” instead of “emotional control” with higher values indicating lower self-regulation.
- We used MANOVAs instead of the initially planned regression analyses as the better fitting statistical approach. The contents of the hypotheses remained untouched.
- After correlation analyses, we conducted all analysis using only other-reports whenever available (inhibition and emotion regulation), because experimental data for these variables did not show relations with prosocial behavior.
- To derive the SR patterns, we did not only consider T1, but also T2 and T3 data to draw a more comprehensive picture.
